# Breast Education Improves Adolescent Girls' Breast Knowledge, Attitudes to Breasts and Engagement With Positive Breast Habits

**DOI:** 10.3389/fpubh.2020.591927

**Published:** 2020-10-30

**Authors:** Atefeh Omrani, Joanna Wakefield-Scurr, Jenny Smith, Ross Wadey, Nicola Brown

**Affiliations:** ^1^Faculty of Sport Health and Applied Science, St Mary's University, Twickenham, United Kingdom; ^2^Research Group in Breast Health, University of Portsmouth, Portsmouth, United Kingdom; ^3^Department of Sport and Exercise Sciences, University of Chichester, Chichester, United Kingdom

**Keywords:** breast education, breast health, adolescent girls, breast cancer education, bra fit, health education

## Abstract

Many females experience breast-related issues that are considered to negatively impact health and well-being. These include breast cancer, issues related to incorrect bra fit, and issues related to breast movement including an increased incidence of breast pain, breast sag, and embarrassment, which can be a barrier to physical activity participation. Knowledge and awareness of these breast issues among females is low. Furthermore, these breast concerns are more prevalent in adolescent girls compared to adult females, with 87% of girls reporting ≥ one breast concern. This study evaluated the short- and longer-term impact of a 50 min breast education intervention on adolescent girls' (11 to 14 years) breast knowledge, attitudes to breasts and engagement with positive breast habits. A mixed methods, controlled, longitudinal, cohort design was employed, using two control schools (*n*: 412; receiving no intervention) and two intervention schools (*n*: 375; receiving the intervention) from privileged and less privileged areas. Adolescent girls in four schools completed a validated breast survey pre- and immediately post-intervention as well as 3 and 6 months post-intervention. Additionally, in one intervention school, six focus groups were conducted immediately and 4 months after the intervention. The intervention was equally effective in the two intervention schools. Following the intervention, participants in the intervention schools significantly improved their breast knowledge, their attitudes to breasts and their engagement with positive breast habits, compared to participants in the control schools, *p* < 0.01 (with large effect sizes). These improvements were sustained 6 months post-intervention. Participants described the session as “informative,” it made them “feel less embarrassed” about their breasts; they also reported wanting to do more exercise and to change their bra purchasing and bra wearing habits. These novel, positive findings provide insight into the benefits of teaching adolescent girls about breasts from a young age and can be used to inform effective breast education in schools. It is recommended that education on multiple breast topics should be introduced in schools, preferably being first introduced in primary schools, with a modular structure and progressive information.

## Introduction

There are negative factors associated with the breast that are considered influential to the health and well-being of adult and adolescent females (1–4). These include breast cancer, and issues related to independent breast movement and poor bra fit. Despite breast cancer being recognized as an important and serious public health issue, studies indicate that women have inadequate knowledge of breast cancer risk factors and symptoms of breast cancer (4, 5). In addition, the female breast is easily deformed by external forces such as gravity, due to its highly malleable structure (6, 7) and has been reported to move up to 4 cm during walking, to 15 cm when running without breast support (8). This breast movement can cause discomfort and embarrassment (9), exercise-related breast pain (10, 11), and may lead to breast sag (ptosis) due to potential damage of the breast structure (6). These breast-related issues also create a barrier to sports and physical activity participation, negatively affecting women's overall health and well-being (2, 12). An appropriate, well-fitted breast support (such as a sports bra) has been suggested to reduce breast movement (2, 6, 11). As the majority of women are not knowledgeable about bra sizing and fitting, with 70 to 100% of women reported to be wearing the incorrect bra size (13–16), evidence indicates that the breast-related issues described above are more prevalent in adolescents compared to adult females (1–3, 9, 17–19).

Adolescence is a challenging time for girls; breasts and breast development can be embarrassing and confusing, negatively impacting body image, self-esteem (20), and physical activity participation (17). A survey of 2,089 school girls aged 11 to 18 years provided evidence of the need for, and guidance on breast education for this group (17, 18). The majority (87%) of girls surveyed reported ≥ one breast concern such as how to check for breast cancer, breast bounce when exercising, breast pain, what boys think about girls' breasts, and how to find a bra that fits. One in four girls reported negative feelings about their breasts and more than half of girls reported their breasts as a barrier to physical activity participation, compared to one in five adult females (2). However, encouragingly 87% of girls reported wanting to know more about their breasts, with 44% wanting to learn more about breasts specifically related to physical activity.

Females experience numerous breast changes in their lifetime (21) and while most breast conditions are benign, it is important for females to be breast aware; i.e., to know how their breasts look and feel normally so they can detect any changes immediately and seek support if required (22–24). Breast education interventions are recommended as effective public health actions in the prevention and control of breast cancer (23, 25, 26). Adolescence has been recommended as an ideal age to promote breast health and breast awareness, as it is during adolescence that most future health-related lifestyles, behaviors, habits and attitudes are formed (23, 27, 28). Furthermore, when surveying 2,089 girls, the majority (72%) reported wanting to know more about breast cancer, with 69% rating this topic as extremely important (1). Increasing adolescent girls' knowledge of the risks of breast cancer and benefits of early detection may improve breast cancer outcome and survival, and encourage these behaviors in adulthood when breast cancer risk is greater (18, 29). Moreover, adolescent girls' body satisfaction and self-esteem may be improved by teaching them about breast sizes and shapes and how breasts change over time (18).

In addition to education on breast cancer, education on other breast topics is required. Education on breast support and bra fit has been suggested as an effective strategy to reduce breast movement and its associated negative health consequences (2, 6, 11). However, the bra market with its wide choice of brands, styles and sizes can be a confusing place for adolescents, making the selection of appropriate, well-fitted bras difficult (18). The majority of women are not trained in bra sizing and fitting nor have enough knowledge to make bra purchasing decisions (16). Literature suggests that females should be educated on professional bra fitting criteria to improve their ability to independently choose a well-fitted bra (30–33). Improving bra fit can also reduce the negative health outcomes associated with wearing ill-fitting bras such as deep bra furrows on the shoulders caused by excessive strap pressure, neck and back pain, and upper limb neural symptoms (13, 31). Well-designed and correctly fitted sports bras are reported to be more effective in limiting breast movement than standard fashion bras or crop tops, resulting in reduced breast pain, greater comfort and enhanced sporting performance (6, 9, 11, 30, 34). However, studies have shown that adolescent girls' knowledge of breasts, bra fit, and appropriate breast support is relatively low (3, 17, 18, 35). Furthermore, more than half of 2,089 girls surveyed reported never wearing a sports bra during exercise (11). A breast education initiative that promotes the benefits of appropriate breast support (e.g., a sports bra) and incorporates training on bra selection and fit may eliminate the breast as a barrier to physical activity participation in adolescent females (17).

Education on sensitive topics (e.g., HIV/AIDS, cancer prevention) in school settings have been shown to increase adolescents knowledge, and improve attitudes and behaviors (36–38), demonstrating the benefits of school based health education. However, the curriculum in many countries, including the UK and the US, do not offer compulsory breast education beyond the biology of puberty (39, 40). Clark et al. (41) and Horton (42) recommend that breast education programmes address adolescents attitude barriers to breasts (e.g., embarrassment, negative feelings about breasts), in addition to addressing knowledge limitations (41, 42). Furthermore, research suggests that breast education programmes should cover multiple topics, including, but not limited to; breast awareness, breast sag, breast pain, breast size and breast changes, appropriate breast support and bra fit (18). However, previous breast education studies have focused on breast cancer and breast self-examination only in adolescent populations (35, 41–43). To date, only one education study has focused on other aspects related to the breasts such as breast movement, bra fit and appropriate breast support, though this focused on an athletic cohort (44).

Educating adolescent girls from a young age about a wide range of breast topics is likely to reduce breast-related concerns, improve attitudes to breasts and breast issues, and promote positive breast habits in adolescent girls (e.g., checking breasts, wearing a sports bra). It has been reported that the most appropriate age at which breast topics should be introduced is 11 years, which is the average age of breast budding in all ethnic groups (8.5 to 13.3 years) (45, 46). Currently, breast education studies have focused on individual breast topics in isolation and have primarily assessed improvements in knowledge. No studies have incorporated education on multiple breast topics, or assessed attitudes to breasts (e.g., embarrassment, feelings about breasts), and engagement with positive breast habits. Furthermore, no qualitative data have been collected in breast education studies to provide in-depth understanding of the impact of a breast education intervention (35, 41–43). Therefore, using a mixed methods design, the primary aim of this study is to evaluate the short- and longer-term impact of a breast education intervention on adolescent schoolgirls' breast knowledge, attitudes to breasts, and engagement with positive breast habits. Additionally, the study aims to establish whether the intervention would be effective in schools of differing area-level deprivation and socio-economic status. In addition to collecting quantitative data, focus groups were used to obtain qualitative data to gain rich insight and in-depth understanding into the effectiveness and importance of the breast education intervention (47–49). The unique emotional and cognitive characteristics of early adolescence include a strong desire to be with one's peers and a preference for group rather than one-on-one activities (50); making focus groups a developmentally appropriate qualitative method for use with this population (51, 52).

## Methodology

### Study Design and Study Population

To evaluate the impact of the breast education intervention, a mixed methods, controlled, longitudinal, cohort study was employed, utilizing a valid and reliable breast survey (53) together with focus groups.

Sample size calculation with the following: α = 0.05; two-tailed test; power = 0.80; effect size = 0.50 (medium) indicated that the study needed a minimum of 128 participants, with 64 in each group. Following institutional ethical approval, two single-sex control schools (control groups; *n* = 375) and two single-sex intervention schools (intervention groups; *n* = 412) were recruited via a convenience sampling method. To increase the representativeness of the sample (54) and establish the effectiveness of the intervention in areas of differing area-level deprivation and socio-economic status (55), one intervention and control group (intervention-1 and control-1) were recruited from privileged areas in London, UK, with the other intervention and control group (intervention-2 and control-2) recruited from less privileged areas in London, UK. All participating schools selected an opt-out method for parent/guardian consent, whereby parents who did not wish their child to participate were required to return the consent form. All adolescent girls who participated in the study also gave informed consent. In total, 787 adolescent girls aged 13.2 ± 0.8 (range 11 to 14) years took part in the study. Of these, 40.4% were Black/African/Caribbean/Black British, 23% were Asian/Asian British, 18.7% were White, 9.3% were Mixed/multiple ethnic groups, 3% were from other ethnic backgrounds and 5.6% did not report their ethnic group (Table 1).

**Table 1:** Participants' mean age (± standard deviation) and ethnicity (%) in four groups.

**Variable**	**Intervention-1 *n*: 129**	**Control-1 *n*: 121**	**Intervention-2 *n*: 246**	**Control-2 *n*: 291**
Age (years)	13.3 ± 0.3	12.3 ± 0.2	13.4 ± 0.9	13.3 ± 0.9
**Ethnicity**				
White	62.8%	19.8%	8.5%	7.2%
Asian/Asian British	20.2%	62%	8.5%	20.3%
Mixed/multiple ethnic groups	11.6%	9.9%	6.9%	10.0%
Black/African/ Caribbean/Black British	3.1%	0.0%	63%	54.6%
Other ethnic group	1.6%	1.7%	2%	5.2%
Did not report their ethnic group	0.8%	6.6%	11%	2.7%

### Breast Education Intervention

The breast education intervention was developed externally by breast health experts based on evidence obtained from the target population (17, 18) and literature on effective pedagogy for education on sensitive topics (35, 56, 57). The intervention was designed for adolescents aged 11 to 14 years. The intervention consisted of group discussions and a PowerPoint slide presentation covering a broad content including names for breasts, breast anatomy, causes of and preventing breast bounce, breast pain and breast sag, breast size and breast changes, appropriate breast support, bra sizing and bra fit, breast awareness and signs of breast cancer. Short videos were also included demonstrating the breast movement in a daily bra and a sports bra and explaining professional bra fit criteria. The education intervention was designed to take ~50 min to deliver and was delivered by female PSHE teachers and male science teachers. To promote consistency of delivery, a detailed lesson plan and full instructions/activities were provided.

### Breast Education Intervention Evaluation

#### Breast Survey

**Figure 1:**
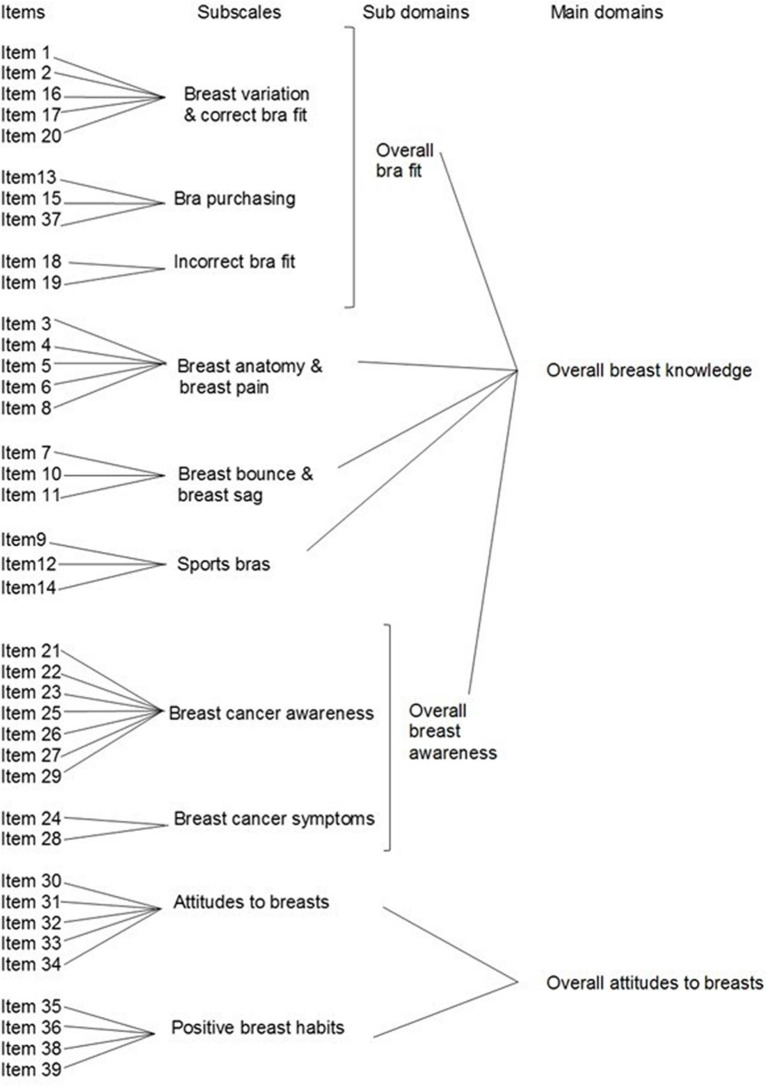
Breast survey items (*n* = 39), subscales (*n* = 10), and summary measures. The subscales are collapsed into an overall breast knowledge, overall attitudes to breasts (main domains), overall bra fit, and overall breast awareness (sub domains). *Measures breast knowledge (*n*: 11). ^†^Measures attitudes to breasts and engagement with positive breast habits (*n*: 3).

Adolescent girls' breast knowledge, attitudes to breasts and engagement with positive breast habits were measured using a 39-item valid and reliable breast survey (53, 58) that was designed in parallel with the development of the breast education intervention. The survey was designed with consideration of adolescents' developmental stage and underwent extensive evaluation to establish the validity and reliability of the survey, as detailed in Omrani et al. (53). The breast survey consists of two main domains (overall breast knowledge and overall attitudes to breasts), two sub domains (overall bra fit knowledge and overall breast awareness knowledge) and 10 individual subscales (Figure 1). Each subscale addresses a specific breast concern which is consistent with the breast needs of adolescent girls (1, 17, 18) and aligns with the content of the breast education intervention. Items utilized a Likert scale format with four possible responses for knowledge items (“completely false,” “sort of false,” “sort of true,” and “completely true”) and attitude and habit items [“fully disagree,” “mainly disagree,” “mainly agree,” and “fully agree” (53, 58)]. Item scores ranged from one (lowest) to four (highest), with negatively worded items reverse scored.

**Figure 2:**
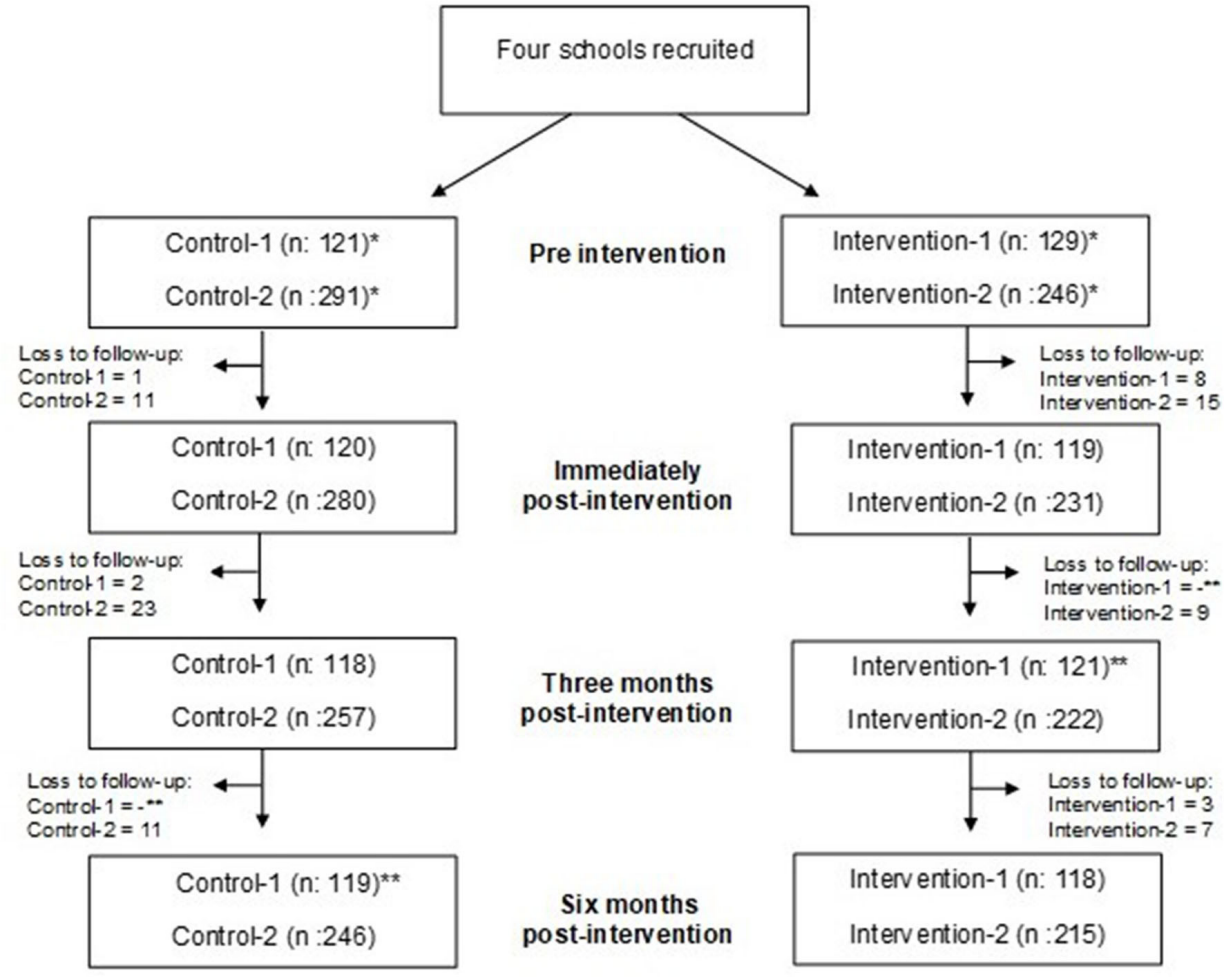
Flow chart of study participants in control and intervention groups at four time points. *Study final sample size: Missing data was dealt with using hot-deck imputation. **Participants missed a particular follow-up, and then returned in the study at the next follow.

To evaluate the short- and longer-term impact of the intervention, participants in all four groups completed the breast survey 1-week pre-intervention and immediately post-intervention (i.e., 5 to 15 min after the delivery of the breast education session), as well as 3 and 6-months post-intervention. Participants in the intervention groups completed the breast survey electronically (47.6%) and participants in the control groups completed the breast survey on paper (52.3%) due to computer access issues. To increase reliability, the items and format of the electronic and paper-based surveys were identical (59). Participants were lost to follow-up during the study (Figure 2), which is a common problem in longitudinal studies (60–62). For this study, the loss to follow-up rate was 11.4% (*n*: 90; 88.6% follow-up/retention rate) in which participants had missing data at one (*n*: 79) or two time points (n: 11), which is an acceptable follow-up thresholds for longitudinal cohort studies (63). Hot-deck imputation, which is a reliable and common method for handling missing data, was used to replace missing data in this study to produce a more complete dataset that is not adversely biased (64, 65).

#### Focus Groups

Focus groups were used to obtain qualitative data; three focus groups (*n*: 23 in total) were conducted in one intervention group immediately post-intervention, and three focus groups (*n*: 21 in total) were conducted with the same participants ~4 months post-intervention. Of the participants, 52.1% were Black/African/Caribbean/Black British, 26% were White, 13% were Asian/Asian British, and 8.9% were Mixed/multiple ethnic groups.

Each focus group consisted of adolescent girls from the same school year to maintain some degree of homogeneity, capitalize on participants' shared experience (48, 66), and build rapport between group members (67). This also helped to approach discussions from different perceptions and experiences as it increased the likelihood that girls were at different stages of breast development. Of the 23 participants that took part in the focus groups immediately post-intervention, eight participants were in year 7 (11 to 12 years), nine were in year 8 (12 to 13 years) and six were in year 9 (13 to 14 years). Focus groups 4 months post-intervention were conducted in the following academic year; therefore, all participants were in a higher year (*n*: 21; two participants were absent). All focus groups were conducted in a classroom within the participants' school, and a semi-structured format was utilized. The duration of focus groups ranged from 45 to 50 min and were audio recorded. Following each focus group, the recordings were transcribed verbatim to maintain purity of data and enhance the truth value of the research (68).

### Analysis

#### Breast Survey—Comparison Between Intervention and Control Groups Over Time

Gain scores (difference between post-tests and pre-test) were used to take account of chance imbalances between groups and compare distinct groups in terms of their average gain over time (69, 70). To compare groups over time, a two-way mixed MANOVA assessed changes in mean gain scores of the main domains (*n*: 2), sub-domains (*n*: 2) and individual subscale scores (*n*: 10) between control and intervention groups. Three gain scores were calculated; pre to immediately post-intervention, pre to 3-months post-intervention, and pre to 6-months post-intervention. The significance level was set at 0.01 to control for type-I error and to correct and compensate for the violation of homogeneity of variances. A significant multivariate interaction effect was followed by testing simple effects in each of the variables separately (simple effects for groups). The mean scores for each group in separate ANOVA's were compared using the Bonferroni correction to compensate the inflated family-wise error rate (α) due to multiple comparisons (71).

#### Breast Survey—Individual Group Analysis

A one-way repeated measures MANOVA was performed to assess how the mean scores of the main domains (*n*: 2), sub domains (*n*: 2) and individual subscales (*n*: 10) changed over time in each group. The significance level was set at 0.05. A statistically significant multivariate interaction effect was followed by univariate analysis (one-way repeated measures ANOVAs) to assess the effect of time of testing on each dependent variable separately. Again, to compensate for family-wise error due to multiple comparisons, a Bonferroni correction was applied. Partial η2 was calculated for each test; with effect sizes considered small (0.01), medium (0.06), and large (≥0.14) (59).

#### Focus Group Analysis

Focus group data were thematically analyzed following the framework method (72, 73). To show commonality of themes among participants and establish the pattern of data, the prevalence of certain themes were determined (74, 75). The analysis was carried out using both inductive and deductive approaches, concurrently, but with different dominancy, hence, it was “deductive-dominant” (76). This approach allowed comprehensive analysis of the data and no data was excluded on the basis that it did not “fit” a certain theme. The analysis was conducted by the lead author; however, to enhance credibility and validity of the findings, a breast health expert with qualitative analysis experience verified the focus group transcriptions and themes to triangulate the data (77). The breast health expert acted as a “critical friend” who provided critical feedback, asked provocative questions and challenged interpretations to encourage reflection, enhancing rigor of the qualitative research (78, 79).

## Results

### Breast Survey—Comparison Between Intervention and Control Groups Over Time

The mean gain scores were significantly higher in the intervention groups compared to the control groups, *p* < 0.01, with mean gain scores ranging from 0.23 to 1.25 (out of a maximum positive score of 4 or negative score of −4) in the intervention groups, and −0.08 to 0.03 in the control groups (Table 2). There were no significant differences observed in the mean gain scores between the control groups, or between the intervention groups, *p* > 0.01. Simple effects analysis for groups confirmed the above results; for each dependent variable (*n*: 14), the mean gain scores at each category of time were significantly higher in the intervention groups compared to the control groups, *p* < 0.01. The mean gain scores did not significantly differ between the control groups, or between the intervention groups, with the exception of subscale three (“incorrect bra fit”) where mean gain scores were significantly higher in intervention-1 compared to intervention-2 (pre to 6-months post-intervention), and subscale five (“breast bounce and breast sag”) where mean gain scores were significantly lower in intervention-1 compared to intervention-2 (pre to 3-months post-intervention) (Table 2).

**Table 2:** Mean and standard deviation (SD) gain scores for the main domains, subdomains, and individual subscales for four groups at all time points.

**Measure**	**Time point**	**Intervention-1**		**Control-1**		**Intervention-2**		**Control-2**	
		**Mean**	**SD**	**Mean**	**SD**	**Mean**	**SD**	**Mean**	**SD**
**Main domains**									
Overall breast knowledge	Pre to immediately post-intervention	0.71^*^	0.30	0.00	0.20	0.81^*^	0.37	0.00	0.36
	Pre to 3-months post-intervention	0.60^*^	0.28	0.00	0.22	0.72^*^	0.38	−0.02	0.32
	Pre to 6 months post-intervention	0.54^*^	0.30	0.01	0.32	0.58^*^	0.39	−0.03	0.34
Overall attitudes to breasts	Pre to immediately post-intervention	0.44^*^	0.50	0.00	0.24	0.36^*^	0.39	−0.02	0.47
	Pre to 3-months post-intervention	0.59^*^	0.50	0.06	0.43	0.54^*^	0.50	−0.03	0.55
	Pre to 6-months post-intervention	0.47^*^	0.56	0.03	0.37	0.45^*^	0.60	−0.01	0.56
**Sub domains**									
Overall bra fit	Pre to immediately post-intervention	0.76^*^	0.36	0.00	0.28	0.74^*^	0.43	0.00	0.41
	Pre to 3-months post-intervention	0.64^*^	0.36	0.00	0.31	0.67^*^	0.45	−0.03	0.39
	Pre to 6-months post-intervention	0.57^*^	0.36	0.01	0.39	0.47^*^	0.46	−0.02	0.42
Overall breast awareness	Pre to immediately post-intervention	0.72^*^	0.41	0.00	0.29	0.74^*^	0.52	−0.01	0.51
	Pre to 3-months post-intervention	0.59^*^	0.39	−0.01	0.37	0.69^*^	0.51	0.02	0.49
	Pre to 6-months post-intervention	0.50^*^	0.45	−0.03	0.41	0.51^*^	0.54	0.00	0.53
**Subscales**									
S1-Breast variation and correct bra fit	Pre to immediately post-intervention	0.79^*^	0.51	0.03	0.39	0.76^*^	0.50	0.01	0.54
	Pre to 3-months post-intervention	0.71^*^	0.50	0.03	0.43	0.69^*^	0.52	−0.03	0.49
	Pre to 6-months post-intervention	0.70^*^	0.49	0.07	0.47	0.57^*^	0.57	−0.03	0.52
S2-Bra purchasing	Pre to immediately post-intervention	0.46^*^	0.54	−0.06	0.36	0.44^*^	0.65	−0.08	0.67
	Pre to 3-months post-intervention	0.29^*^	0.59	−0.06	0.41	0.44^*^	0.65	−0.08	0.67
	Pre to 6-months post-intervention	0.23^*^	0.62	−0.05	0.51	0.41^*^	0.67	0.00	0.67
S3-Incorrect bra fit	Pre to immediately post-intervention	1.05^*^	0.70	0.02	0.77	1.05^*^	0.80	−0.03	0.78
	Pre to 3-months post-intervention	0.96^*^	0.72	−0.04	0.78	0.99^*^	0.80	−0.02	0.87
	Pre to 6-months post-intervention	0.80^*^^†^	0.76	−0.04	0.85	0.39^*^	0.93	−0.04	0.81
S4-Breast anatomy and breast pain	Pre to immediately post-intervention	0.70^*^	0.46	−0.02	0.38	0.91^*^	0.52	0.00	0.53
	Pre to 3-months post-intervention	0.58^*^	0.47	0.04	0.43	0.76^*^	0.53	−0.02	0.51
	Pre to 6-months post-intervention	0.57^*^	0.46	0.04	0.48	0.69^*^	0.51	−0.07	0.52
S5-Breast bounce and breast sag	Pre to immediately post-intervention	0.82^*^	0.59	0.03	0.59	1.09^*^	0.59	−0.05	0.59
	Pre to 3-months post-intervention	0.70^*^	0.60	0.08	0.61	1.00^*^^†^	0.61	0.01	0.70
	Pre to 6-months post-intervention	0.67^*^	0.62	0.09	0.69	0.94^*^	0.70	0.02	0.75
S6-Sports bras	Pre to immediately post-intervention	0.50^*^	0.55	0.05	0.42	0.74^*^	0.55	−0.07	0.63
	Pre to 3-months post-intervention	0.44^*^	0.53	−0.03	0.53	0.68^*^	0.57	−0.05	0.57
	Pre to 6-months post-intervention	0.33^*^	0.57	0.04	0.58	0.54^*^	0.65	−0.05	0.61
S7-Breast awareness	Pre to immediately post-intervention	0.60^*^	0.45	0.00	0.32	0.65^*^	0.56	−0.02	0.53
	Pre to 3-months post-intervention	0.46^*^	0.44	0.03	0.42	0.59^*^	0.56	0.03	0.54
	Pre to 6-months post-intervention	0.40^*^	0.48	−0.04	0.47	0.48^*^	0.59	−0.02	0.60
S8-Breast cancer symptoms	Pre to immediately post-intervention	1.25^*^	0.70	0.04	0.54	1.09^*^	0.75	−0.02	0.83
	Pre to 3-months post-intervention	1.07^*^	0.69	−0.02	0.74	1.03^*^	0.74	−0.03	0.90
	Pre to 6-months post-intervention	0.91^*^	0.74	0.00	0.73	0.59^*^	0.85	0.00	0.86
S9-Attitudes to breasts	Pre to immediately post-intervention	0.23^*^	0.59	−0.03	0.33	0.31^*^	0.52	−0.06	0.63
	Pre to 3-months post-intervention	0.45^*^	0.53	−0.10	0.57	0.37^*^	0.62	−0.07	0.68
	Pre to 6-months post-intervention	0.28^*^	0.63	−0.01	0.48	0.33^*^	0.73	−0.04	0.69
S10-Positive breast habits	Pre to immediately post-intervention	0.69^*^	0.72	0.05	0.38	0.44^*^	0.51	−0.01	0.63
	Pre to 3-months post-intervention	0.79^*^	0.72	0.04	0.61	0.80^*^	0.69	0.00	0.74
	Pre to 6-months post-intervention	0.74^*^	0.71	0.10	0.61	0.57^*^	0.80	0.01	0.73

*S, subscale*.

* *significant difference compared to control group, p < 0.01*.

† *significant difference compared to intervention group, p < 0.01*.

### Breast Survey—Individual Group Analysis

**Figure 3:**
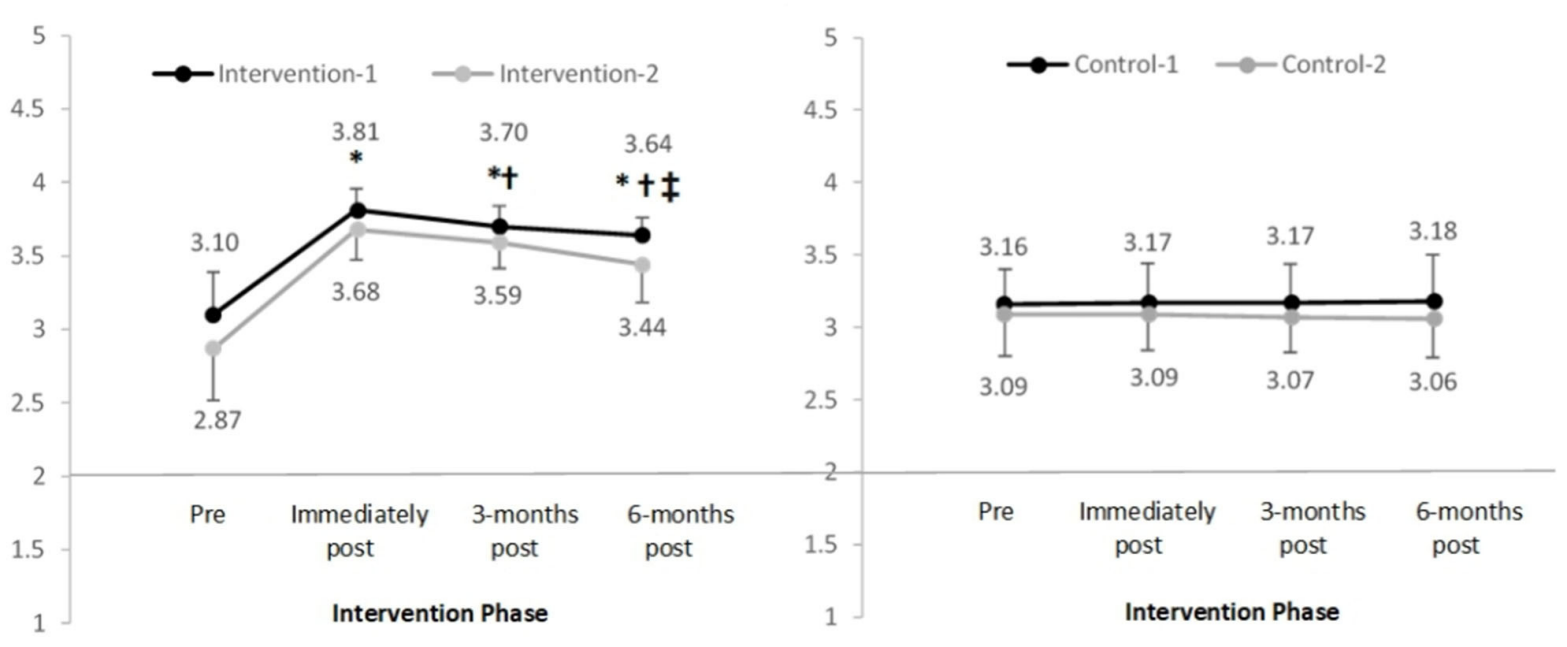
Mean and standard deviation of the main domain “overall breast knowledge” in four groups over time (range 1–4). *Significant difference compared to pre-intervention, *p* < 0.025; ^†^Significant difference compared to previous time-point, *p* < 0.025; ^‡^Significant difference between immediately to 6-months post-intervention, *p* < 0.025.

**Figure 4:**
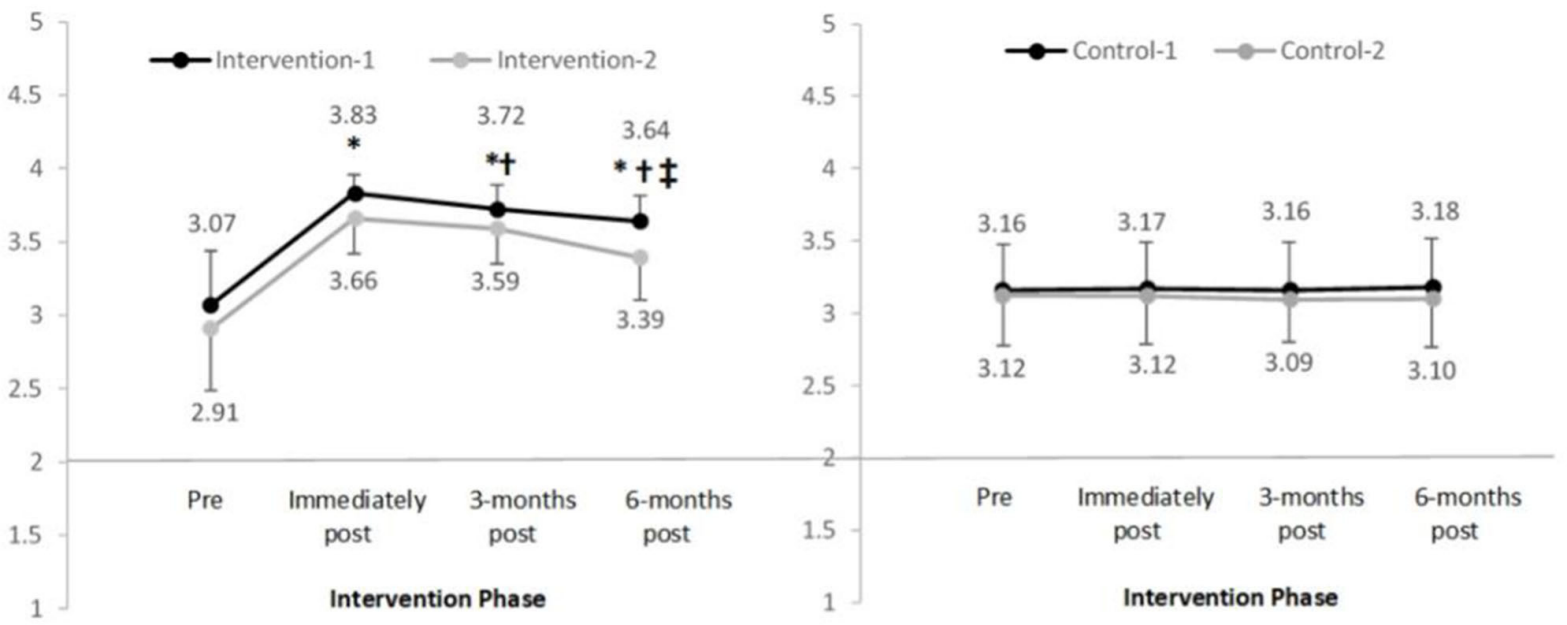
Mean and standard deviation of the sub-domain “overall bra fit” in four groups over time (range 1–4). *Significant difference compared to pre-intervention, *p* < 0.025; ^†^Significant difference compared to previous time-point, *p* < 0.025; ^‡^Significant difference between immediately to 6-months post-intervention, *p* < 0.025.

**Figure 5:**
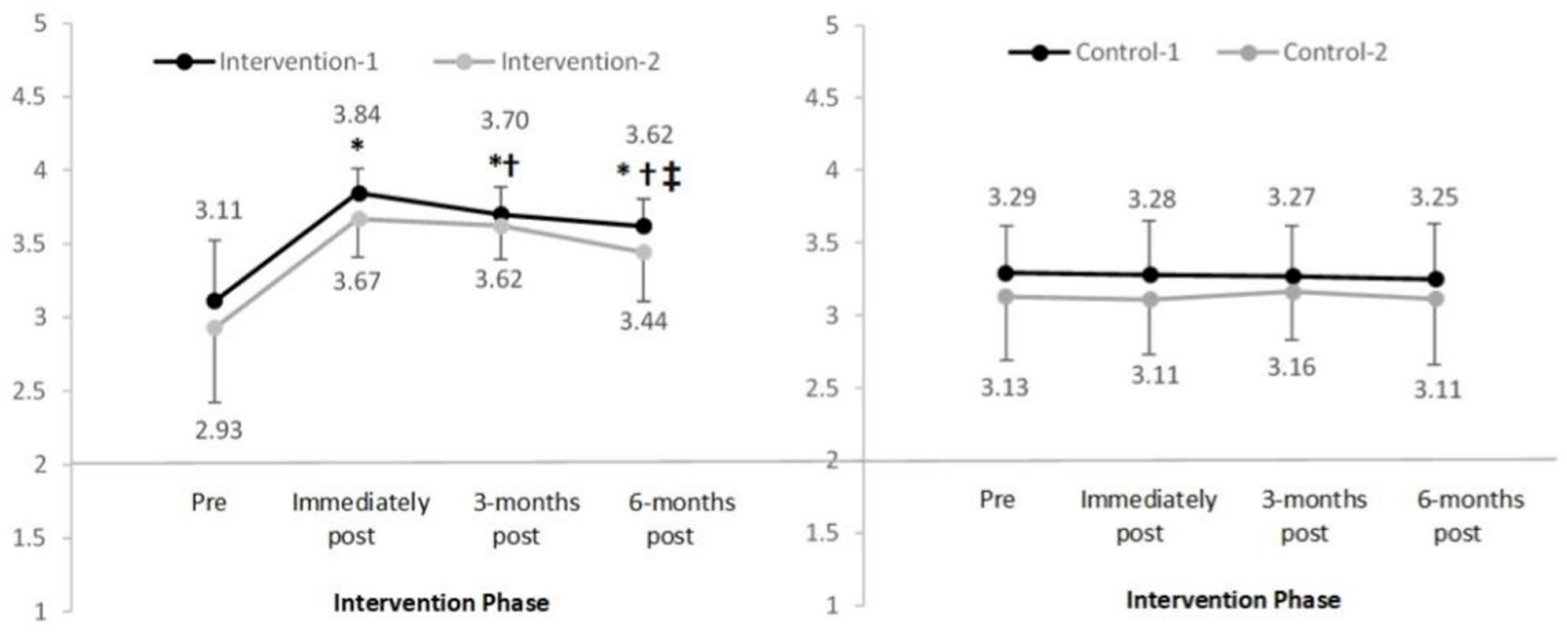
Mean and standard deviation of the sub-domain “overall breast awareness” in four groups over time (range 1–4). *Significant difference compared to pre-intervention, *p* < 0.025; ^†^Significant difference compared to previous time-point, *p* < 0.025; ^‡^Significant difference between immediately to 6-months post-intervention, *p* < 0.025.

When examining the main domains (Figure 3), sub-domains (Figures 4, 5), and subscales (Table 3) that assessed knowledge, significant increases were observed in the intervention groups scores pre to post-intervention. Mean increases ranged from 0.23 to 1.24, *p* < 0.05 with large effect sizes observed (Table 4). Mean knowledge scores then reduced over time, although at all subsequent time points (3- and 6-months post-intervention) remained significantly higher compared to pre-intervention, *p* < 0.05.

**Table 3:** Mean and standard deviation (SD) scores for the 10 individual subscales for four groups at all time points.

**Subscale**	**Time point**	**Intervention-1**		**Control-1**		**Intervention-2**		**Control-2**	
		**Mean**	**SD**	**Mean**	**SD**	**Mean**	**SD**	**Mean**	**SD**
**Knowledge subscales**									
S1-Breast variation and correct bra fit	Pre intervention	3.09	0.48	3.21	0.39	2.97	0.49	3.17	0.45
	Immediately post-intervention	3.89^*^	0.17	3.24	0.40	3.74^*^	0.26	3.19	0.48
	3-months post-intervention	3.81^*^^†^	0.19	3.25	0.46	3.66^*^^†^	0.27	3.14	0.39
	6-months post-intervention	3.79^*^^‡^	0.21	3.28	0.44	3.54^*^^†^^‡^	0.34	3.12	0.48
S2-Bra purchasing	Pre intervention	3.28	0.52	3.45	0.41	3.05	0.61	3.32	0.57
	Immediately post-intervention	3.75^*^	0.23	3.39	0.46	3.57^*^	0.37	3.24	0.55
	3-months post-intervention	3.58^*^^†^	0.37	3.39	0.41	3.50^*^^†^	0.39	3.24	0.46
	6-months post-intervention	3.51^*^^‡^	0.37	3.40	0.45	3.47^*^^†^^‡^	0.45	3.32	0.50
S3-Incorrect bra fit	Pre intervention	2.72	0.71	2.65	0.70	2.58	0.77	2.72	0.73
	Immediately post-intervention	3.78^*^	0.38	2.68	0.72	3.63^*^	0.47	2.70	0.74
	3-months post-intervention	3.69^*^	0.33	2.61	0.69	3.57^*^	0.39	2.71	0.67
	6-months post-intervention	3.53^*^^†^^‡^	0.38	2.61	0.70	2.97^*^^†^^‡^	0.69	2.69	0.61
S4-Breast anatomy and breast pain	Pre intervention	3.12	0.44	2.99	0.38	2.81	0.48	3.08	0.44
	Immediately post-intervention	3.82^*^	0.21	2.97	0.37	3.73^*^	0.29	3.09	0.43
	3-months post-intervention	3.71^*^^†^	0.25	3.03	0.37	3.58^*^^†^	0.28	3.05	0.41
	6-months post-intervention	3.69^*^^‡^	0.19	3.02	0.47	3.50^*^^‡^	0.33	3.00	0.43
S5-Breast bounce and breast sag	Pre intervention	2.98	0.56	2.98	0.50	2.51	0.56	2.78	0.59
	Immediately post-intervention	3.81^*^	0.25	3.02	0.52	3.61^*^	0.39	2.73	0.62
	3-months post-intervention	3.68^*^^†^	0.29	3.06	0.54	3.52^*^	0.36	2.80	0.57
	6-months post-intervention	3.66^*^^‡^	0.29	3.07	0.57	3.45^*^^‡^	0.46	2.81	0.60
S6-Sports bras	Pre intervention	3.31	0.49	3.33	0.45	2.99	0.52	3.25	0.50
	Immediately post-intervention	3.81^*^	0.25	3.37	0.44	3.74^*^	0.31	3.18	0.54
	3-months post-intervention	3.75^*^	0.27	3.29	0.49	3.68^*^	0.33	3.20	0.45
	6-months post-intervention	3.65^*^^‡^	0.29	3.38	0.47	3.54^*^^†^^‡^	0.42	3.19	0.50
S7-Breast awareness	Pre intervention	3.32	0.46	3.49	0.34	3.11	0.59	3.36	0.47
	Immediately post-intervention	3.92^*^	0.12	3.47	0.41	3.76^*^	0.25	3.32	0.40
	3-months post-intervention	3.79^*^^†^	0.19	3.52	0.40	3.70^*^^†^	0.23	3.39	0.36
	6-months post-intervention	3.72^*^^‡^	0.20	3.44	0.42	3.60^*^^‡^	0.36	3.33	0.50
S8-Breast cancer symptoms	Pre intervention	2.39	0.62	2.61	0.63	2.32	0.62	2.37	0.66
	Immediately post-intervention	3.63^*^	0.44	2.65	0.56	3.40^*^	0.56	2.34	0.67
	3-months post-intervention	3.46^*^^†^	0.46	2.58	0.60	3.35^*^^†^	0.47	2.36	0.72
	6-months post-intervention	3.30^*^^‡^	0.42	2.60	0.63	2.91^*^^†^^‡^	0.64	2.39	0.71
**Attitude and habits subscales**									
S9-Attitudes to breasts	Pre intervention	3.09	0.56	3.16	0.45	2.88	0.68	2.98	0.65
	Immediately post-intervention	3.32^*^	0.55	3.13	0.48	3.20^*^	0.61	2.91	0.51
	3-months post-intervention	3.54^*^^†^	0.35	3.05	0.51	3.26^*^^†^	0.57	2.91	0.42
	6-months post-intervention	3.37^*^^†^	0.39	3.14	0.54	3.22^*^	0.60	2.93	0.58
S10-Positive breast habits	Pre intervention	2.50	0.68	2.41	0.63	2.43	0.65	2.72	0.66
	Immediately post-intervention	3.20^*^	0.52	2.47	0.60	2.88^*^	0.56	2.74	0.60
	3-months post-intervention	3.30^*^^†^	0.49	2.46	0.59	3.24^*^^†^	0.53	2.74	0.44
	6-months post-intervention	3.09^*^	0.56	2.52	0.62	3.00^*^^†^	0.65	2.75	0.59

*S, subscale*.

* *Significant difference compared to pre-intervention, p < 0.025*.

† *Significant difference compared to previous time-point, p < 0.025*.

‡ *Significant difference between immediately to 6-months post-intervention, p < 0.025*.

**Table 4:** Effect sizes (Partial η2*) related to change over time in the intervention groups.

**Measure**	**Partial** ***η2***	
	**Intervention-1**	**Intervention-2**
**Main domains**		
Overall breast knowledge	0.78	0.71
Overall attitudes to breasts	0.39	0.31
**Sub-domains**		
Overall bra fit	0.73	0.60
Overall breast awareness	0.64	0.53
**Subscales**		
S1-Breast variation and correct bra fit	0.63	0.54
S2-Bra purchasing	0.22	0.25
S3-Incorrect bra fit	0.56	0.45
S4-Breast anatomy and breast pain	0.57	0.61
S5-Breast bounce and breast sag	0.53	0.60
S6-Sports bras	0.32	0.44
S7-Breast awareness	0.51	0.44
S8-Breast cancer symptoms	0.61	0.45
S9-Attitudes to breasts	0.19	0.12
S10-Positive breast habits	0.42	0.33

*S, subscale*.

**Figure 6:**
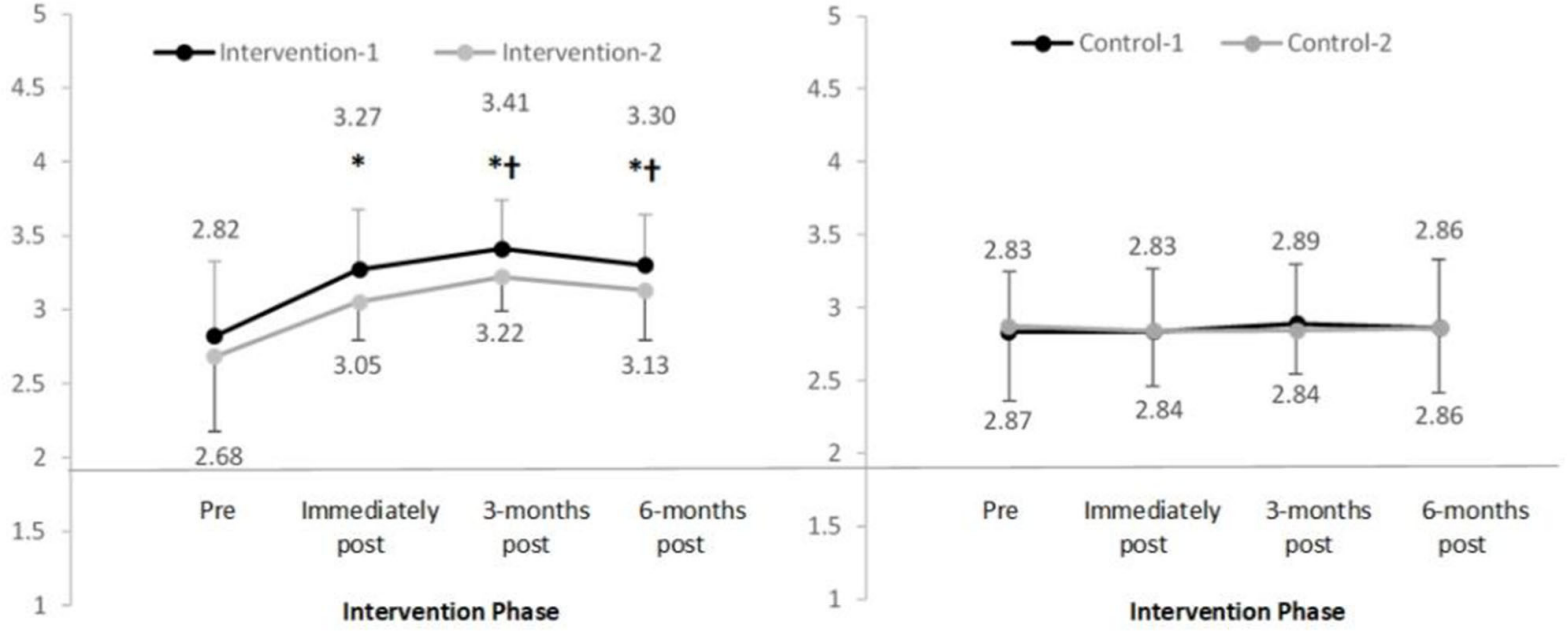
Mean and standard deviation of the main domain “overall attitudes to breasts” in four groups over time (range 1–4). *Significant difference compared to pre-intervention, *p* < 0.025; ^†^Significant difference compared to previous time-point, *p* < 0.025.

In the intervention groups, the mean scores for the main attitude domain (Figure 6) and subscales that measured attitudes to breasts and positive breast habits (Table 3) significantly increased pre to post-intervention. Mean increases ranged from 0.23 to 0.80, *p* < 0.05 with large effect sizes observed (Table 4). Further significant increases in the mean scores were observed immediately post-intervention to 3-months post-intervention, *p* < 0.05. Although mean scores decreased from 3 to 6 months post-intervention, scores remained significantly higher compared to pre-intervention, *p* < 0.05. In the control groups, one-way repeated measures MANOVA indicated no significant change in the mean scores over time in any of the main domains, sub-domains or subscales, *p* > 0.05.

### Focus Group Findings Immediately Post-intervention

Participants were allocated a number from 1 to 23, with numbers 1 to 8 representing participants in the year 7 focus group (Y7), numbers 9 to 17 representing those in the year 8 focus group (Y8) and numbers 18 to 23 representing participants in the year 9 focus group (Y9). The abbreviations Y7, Y8, and Y9 are used to clarify the nature of focus groups. Following the iterative analysis of data obtained from the focus groups, four core themes emerged as explained below (Table 5).

**Table 5:** Themes for the six focus groups.

**Themes**	
**Immediately post-intervention:** 1. Immediate advances in knowledge 2. Time for a positive change 3. Rethinking attitudes to breasts 4. Girls' perception of the session	**~** **Four months post-intervention:** 1. Knowledge retention 2. Applying the knowledge gained 3. The intervention moving forward

#### Theme 1. Immediate Advances in Knowledge

Across all focus groups, participants confirmed that their breast knowledge had increased. Prior to the education session, participants described their breast knowledge as “very poor.” However, after the session participants described their breast knowledge as “very good” and “much more than it was before.” Words and phrases repeatedly used by participants to describe the session were “very good,” “informative,” “helpful,” “important,” “useful,” “interesting,” “new experience,” “opened my eyes,” “really insightful,” and “appropriate.” Participants felt that before the session they were “gullible” and easily believed “rumors” and “myths” about breasts. However, the session made them recognize that “there's a different story” and “the myths are really stupid and do not make any sense” because they have “true knowledge about breasts.” P23-Y9 said “Before, it [breast knowledge] was quite bad and now it's [breast session] like really opened my eyes and I know now a lot more.” This was supported by P19-Y9, “There were stuff that I knew but not enough.”

Following further discussion amongst the groups, it became apparent that increased knowledge about different breast sizes and shapes helped participants to recognize that breast variation is normal:

If your breasts are like two different sizes or like one is big and then the other one is small, you're knowing that it doesn't necessarily mean that you have an illness or something like that, it can mean that it's just natural and you haven't done anything to make your boobs like that, it is not an illness, it's normal. (P15-Y8)

Participants also increased their knowledge about breast bounce, breast sag and breast pain, and reported that this made them realize the importance of wearing appropriate breast support:

I think that it is helpful to know that breasts move 15 cm [during exercise] because if you don't wear a sports bra, like it could be very painful because I had that experience and I think it's helpful to know that it moves 15 cm. (P3-Y7)

In addition, participants reported learning about bras, bra fitting, professional bra fitting criteria and how bra sizes work; this enabled them to explain how a bra should fit properly:

When you wear a bra, like a bra or a sports bra, your breasts should be covered, you have to fit like properly, and if it's very tight or if it's too tight, it may cause your breasts to swell and feel like very sore, and then if it fits like properly it would, like, cause no damage to your breasts. (P2-Y7)

A further topic that participants reported enhanced knowledge of following the session was breast awareness and the importance of checking their breasts. P4-Y7 summarized the topic by saying:

I think another helpful part of the session was where you can see where breast cancer is, like where you check and look for stuff, under your armpits, on your breasts, under your breasts (P8-Y7: under the collar bones). I think it was really helpful just to know. If you don't have it, it's very helpful just in case to see if you have it.

#### Theme 2. Time for a Positive Change

The session positively impacted participants' breast habits. All participants agreed that they were going to make a positive change to their breast habits, for example, checking their breasts, getting fitted for bras (either checking the fit themselves or having a professional bra fit). Participants acknowledged being more breast aware and that they would now check their breasts regularly. The following was a typical conversation about breast awareness that occurred in all focus groups:

I'm going to check [breasts] more often, check for any changes or symptoms or check for a lump and stuff like that because I know how important it is.

Participants also explained that as a result of learning about bra fitting and bra sizes, they will now check if their bra fits properly. Moreover, they stated that they will wear appropriate breast support (e.g., a sports bra) for physical activity, as illustrated in the following quotation:

Usually, I have sports bras at home, but I wouldn't use them but I'm going to now. Whenever I'm like running and my breasts are going up and down, it hurts me a lot because I didn't know about sports bras and stuff, but then I always went through pain when they were going up and down, but now I know that I can use sports bras when I do sports and it would not hurt as much as it did before. (P1-Y7)

In addition to making changes in their own breast habits, participants felt that the session enabled them to pass on knowledge and advice to their peers and family members, as shown below:

I think this session has given me more knowledge that I could pass on to people my age, like find it useful for them, and now that I know this much, I think people would try to appreciate it and that they would know more than they knew before. (P5-Y7)

#### Theme 3. Rethinking Attitudes to Breasts

Learning about breasts and breast issues made participants feel more comfortable and less embarrassed about their breasts and talking about breasts. The majority of participants reported having a more positive attitude toward their breasts. Three participants did not report any changes in their attitudes toward breasts because, encouragingly, they had a positive attitude before, “I was fine before because that's part of my body, I should be proud of my body” (P2-Y9). A sentiment shared by participants in all the focus groups was that they should be proud of their breasts. They also commented that having increased knowledge about breasts enabled them to “understand breasts more” and as a result they reported “feeling more mature,” “less embarrassed” and “more confident and comfortable talking about breasts.” One participant talked about her “flat chest” and that the session had changed her attitudes toward her breasts:

People were just like having big breasts, and I was just there like why are you so flat girl? [talking about herself], and then now I'm just like yep, you are flat, and you are proud. (P3-Y7)

Seven out of nine participants in the Y8 focus group reported that their breasts had some effects on their physical activity participation. They reported that “knowing about breasts” and “doing the right things” can help them to be more physically active and increase their physical activity participation. This topic was only discussed with girls in the Y8 focus group because in the other focus groups there was not enough time to explore this topic.

Some people might not want to do it [physical activity] because they might be embarrassed, especially in sports days when you are running and your boobs are just like all over the place, like touching the floor and coming back. If you know how to prevent that, like by wearing a sports bra, if you know that, you might not be embarrassed. (P11-Y8)

#### Theme 4. Girls' Perception of the Session

Participants appreciated the importance of learning about breasts from a young age and considered the session very helpful. Participants in the Y9 focus group emphasized the importance of the session more than participants in other years:

I thought the session was really insightful because it taught me a lot of things about breasts that I didn't really know about, because when first we did the questionnaire [the breast survey] it talked about, how, what was in the breasts, I didn't know anything about breasts to be honest with you, it really gave me information that I thought I would never know about them. (P19-Y9)

The appropriate age to receive the breast session was also discussed with all focus group participants. Overall, participants considered learning about breasts from a young age very helpful because they can “notice any changes in breasts before it is too late.” The following quotation reflects this view:

I think that it was helpful because when like, if you are like our age, then you need to be aware of this [breast awareness] before you get older. (P3-Y7)

P15-Y8 emphasized that in addition to learning about breast awareness from a young age, it was important to know about breast changes at a young age in order to be aware of the changes that occur during puberty:

Some of us are in the stage that we are transforming from a child to a teenager, because we are changing, we don't really know what is happening and it can be very confusing. Because in primary school we didn't really have that breast education, so yeah, it's important to know at our age.

In general, participants felt that they learnt about breasts at the right age, however, some participants thought that breast education should start earlier, in primary schools (e.g., year 6), as illustrated in the following quotations:

I feel like when you are learning about sex education at the end of year six in primary school, I think you should learn about this [breasts] as well because the sooner that I had more information at that time, I wouldn't be as worried as I am now. (P12-Y8)

### Focus Group Findings 4 Months Post-intervention

Due to the focus groups taking place 4 months post-intervention, all participants had transitioned into the beginning of the next school year. However, for ease of interpretation, the participants continue to be referred to by their initial focus group number (Y7, Y8, and Y9). Three key themes emerged, as detailed below.

#### Theme 1. Knowledge Retention

Overall participants reported that they remembered “most stuff” from the session, but had forgotten some details (e.g., breast cancer symptoms). Participants demonstrated their longer-term knowledge improvement, describing what they had learned about breast sizes, bras, and bra purchasing. Professional bra fitting criteria were also discussed in all the focus groups and participants were able to correctly recall the criteria. All participants also correctly remembered that wearing a well-designed sports bra can help to reduce breast bounce, breast sag and breast pain. Participants in all the focus groups were also able to recall what they learned about breast awareness including some of the signs and symptoms of breast cancer. However, while participants remembered “most of the stuff” from the breast session, half of the participants reported that they have forgotten some details with comments such as “I kind of forgot some of it,” “I forgot some of the signs of breast cancer.” Participants suggested that the session should be delivered “more often,” for example, “twice a year”:

I think you should have them a bit more often, because I think if people, if they learn about it once a year, people might forget it (P9-Y8: yeah, like twice a year) because I forgot quite a bit of it (P10-Y8: yeah). (P13-Y8)

#### Theme 2. Applying the Knowledge Gained

Participants explained how the breast knowledge gained in the session had helped them to have a more positive attitude toward breasts and how it had positively affected their breast habits. For example, participants reported that they checked their breasts after the session, with half continuing to check their breasts regularly and the other half checking “sometimes” and “occasionally”:

I check my breasts every month. I checked my breasts yesterday, I was having a shower and just looked at them in the mirror (P6-Y7: yeah that's what I do). (P7-Y7)

In addition to checking breasts, participants reported practicing other positive breast habits. P1-Y7 shared her experience about a recent bra purchasing with her mother:

There was this time I wanted to go and try to buy a bra, it didn't fit me but I knew it didn't fit me because of the knowledge that I already had so that was quite useful and because my mum didn't know that so now I can teach my mother.

Half of participants reported changing their bra size following the breast session with two thirds of participants also reporting that they started to wear a sports bra when playing sports:

I didn't know if my bra size was right, and then afterwards, I checked it [checked it herself] and I got a new bra basically (P13-Y8 and P11-Y8: yeah, me too). (P15-Y8)

Participants commented that as a result of the breast education intervention, they can now easily talk about their breasts concerns with their mother. P5-Y8 said that~2 months after the session, she had a breast problem, she felt “comfortable” and “talked about it [breasts]” with her mum. Additionally, participants stated that they tried to use their knowledge to “teach others” about breasts:

I talked to my sister about it because she was like kind of being unconformable and stuff, so I talked to her and I was like they [breasts] are ok and I told her if anything goes wrong, she should go and talk to someone about it. (P10-Y8)

In addition to making positive changes to their breast habits, all participants confirmed that they now had a more positive attitude toward their breasts and feel “better” and “more confident” about their breasts. Most of participants reported feeling “proud” about their breasts; stating they were “not embarrassed” and “not ashamed of their breasts” anymore. One participant summarized this by saying:

I feel like I'm bigger, braver. Before I knew about this [breasts], I felt like more a child. Well, I am a child, I'm 12 now, but I feel like I'm a grown up, and I have a lot of possibilities, I can do this, I can do that and I know how to take care of my breasts. (P2-Y7)

Participants recurrently talked about their past experiences in which they “got bullied” or “mocked” because of their very small or large breasts in primary school, mostly in year 6. They also considered changing with boys in the same changing room “very uncomfortable.” There was an agreement among participants that learning about breasts in primary school would help them to deal with these issues better:

If I knew about breasts, I would not feel as insecure because you just know that it's not always going to look like that, it will change. (P12-Y8)

In one of the focus groups (Y8), participants discussed the impact of the session on their sports and physical activity participation. They reported that the knowledge from the session has helped them to “feel more comfortable” when exercising as demonstrated in the following quotation:

I'm taking more care when I'm exercising, like we talked about it before, when I exercise, I actually wear a sports bra because it actually does help. Because I used to find exercise or like going to the gym a bit uncomfortable because my breasts just hurt, but I didn't really understand why, but then afterwards [after the session] I was like ok I actually need to wear a sports bra. (P9-Y8)

#### Theme 3. The Intervention Moving Forward

Despite 4 months having passed since the session was delivered, participants still considered the session very important and useful. Having learned about breasts and applied the knowledge over the 4-month period, participants had a greater insight into the importance of the session compared to immediately post-intervention. All participants recurrently talked about how important it was to learn about breasts and breast cancer “from an early age” because “by learning it at a young age, it can stay in your mind,” “you can notice any changes in your breasts quickly and get it checked out before it's too late.”

Participants perceived the session as very important and suggested that “everyone should know about it [breasts] because everybody has breasts.” They frequently stated that “some people still feel uncomfortable about their breasts; this session would be useful for them.”

Participants in the Y9 focus group reported that they received the session “too late” because their breasts “have started to sag” and “a lot of girls are developing early.” Many participants thought that the best age for learning about breasts is in “year 6 (10 to 11 years).” Participants in the Y9 focus group also provided some insight into the concept of peer-led education and educating parents/guardians about breast health, commenting “I feel like they should get older kids [e.g., year 10 girls] to come to primary schools and educate the kids. (P21-Y9)”

In addition, half of participants reported that “boys should learn about breasts too,” as highlighted in the quotation below:

I feel like everyone should be educated on this if that makes sense, not only us because a lot of times the people who are doing the mocking aren't the girls, it's the guys, and I feel like it's because they don't understand. (P8-Y7)

## Discussion

### Impact of the Intervention on Breast Knowledge

The quantitative results of this study indicated that the breast education intervention was successful in improving adolescent girls' breast knowledge in multiple breast topics. Significant increases in the mean scores of all knowledge related domains, sub-domains and subscales were observed in the intervention groups following the intervention. Additionally, the results indicated that that the intervention was equally effective in the two intervention groups, despite the differences in their area-level deprivation and socio-economic status. Furthermore, when comparing the mean gain scores, these were significantly higher in the intervention groups, compared to the control groups. Comparison to other breast education studies with relatively similar scoring systems (e.g., score range 1 to 3 and 1 to 8) indicated that the mean increases in the current study were comparable, if not larger. For example, the mean increase in knowledge observed in studies by Tanjasiri et al. (80) and Clark et al. (41) were 0.17 (maximum score = 3) and 0.80 (maximum score = 8), respectively. In the current study, the mean increases ranged from 0.23 to 1.24 (maximum score of 4). Therefore, the breast education intervention was deemed successful and effective in increasing girls' knowledge of breast topics with very large effect sizes. The findings from the focus groups also supported the quantitative results, revealing that the intervention increased participants' breast knowledge over time, establishing the short- and longer-term positive impact of the intervention. Across all focus groups, participants confirmed that they learned many new things about breasts and breast health.

In both intervention groups, participants increased their breast variation, bra fit and sports bra knowledge following the intervention. Knowledge of breast sizes and shapes have practical implications for independently choosing a well-fitted bra, due to the fact that breasts' size, shape, and position change throughout the menstrual cycle and throughout life (81). Across the focus groups, participants reported having more knowledge about bras, bra fitting and how bra sizes work, and they were able to correctly explain how a bra should fit properly using the professional bra fitting criteria. This knowledge is important due to negative health outcomes associated with wearing an ill-fitted bra, namely deep bra furrows, neck and back pain, exercise-related breast pain, discomfort and poor posture (11, 13, 82). Increased knowledge of sports bras is also important because well-designed sports bras are more effective in limiting breast motion and associated breast pain and discomfort when compared to standard fashion bras or crop tops (6, 9, 30, 34).

Participants in the intervention groups also significantly increased their knowledge of breast awareness and “breast cancer symptom,” with focus group participants recognizing the importance of early breast cancer detection. These findings reflect those of previous school-based breast cancer education studies (26, 83). This finding has significant health implications because improving knowledge of breast cancer and its symptoms can promote early detection, hence improving breast cancer outcome and survival (23, 29, 84).

### Impact of the Intervention on Attitudes to Breasts

Quantitative and qualitative findings demonstrated that the breast education intervention was equally effective in improving adolescent girls' attitudes to breasts in both intervention groups over the 6-month period. Mean increases of 0.23 to 0.59 were observed in the “attitudes to breasts” domain and subscale in the intervention groups, with partial η*2* ranging from 0.12 to 0.39 (large effect sizes). These findings are consistent with health promotion literature suggesting that an effective health education intervention can also improve attitudes to health issues (28). For example, studies have identified that breast cancer education can result in significant improvements in attitudes to breast self-examination and early breast cancer detection (41, 85).

The quantitative and qualitative findings of this study provide evidence that the delivery of breast education that focuses on breast sizes, shapes, and how breasts change over time (subscales 1 and 4) can empower girls to accept and feel comfortable with the breast changes they undergo during puberty. Participants reported feeling “better,” “proud,” “more comfortable,” and “less embarrassed” about their breasts following the intervention. Fostering positive attitudes to breasts and breast issues might increase adolescent girls' health and well-being by promoting body satisfaction and self-esteem because well-being and self-esteem are empirically related (86).

The quantitative results suggested that participants' attitudes to sports related breast issues (measured by subscale-9) significantly improved as a result of the breast education intervention. These findings are of importance because previous research has identified that the breast had a negative impact on sport and exercise participation in adolescent girls (17). In line with previous research (17), focus group participants reported that breast pain and embarrassment associated with breast bounce had an impact on their physical activity participation. However, participants also reported that learning about these issues and the benefits of wearing appropriate breast support helped them to “do the right things.”

### Impact of the Intervention on Breast Habits

The intervention was successful in fostering positive breast habits among adolescent girls in the intervention groups, with mean increases ranging from 0.45 to 0.80 in subscale 10 (“positive breast habits”), with large effect sizes observed. An important outcome was participants' engagement with breast awareness activities as a result of the intervention. Focus group participants reported checking their breasts and were able to explain what changes they looked for and where they looked for changes. Furthermore, participants reported talking to someone, especially their mothers, about their breast concerns, which is consistent with breast awareness recommendations that any breast changes should be reported (23, 87). These findings highlight the benefits of teaching girls about breast awareness as these behaviors can positively transfer to adulthood when breast cancer risk is greater (23, 29).

The majority of the focus group participants reported checking their bra fit and/or changing their bras because “it wasn't the right size.” The findings of the current study indicated that participants applied their knowledge in the areas of bras and bra fitting to real life scenarios, which enabled them to make an informed decision to change their bras and independently choose a well-fitted bra.

Another crucial finding of the study was that many participants in the focus groups reported starting to wear a sports bra during sports and exercise. Engaging with sports bra use is a positive change as sports bras are recommended for reducing breast movement and issues associated with this movement such as exercise-related breast pain, breast sag, and embarrassment (6, 11), which are all common breast concerns in adolescent girls (18). As discussed by the focus group participants, “doing the right things when doing sports,” such as improving bra fit and the level of breast support worn (e.g., wearing a sports bra), can encourage greater physical activity and sports participation. Research has identified that greater breast knowledge is positively related to sports bra use and, consequently, positively related to levels of physical activity (2).

### The Intervention Moving Forward

Although mean scores in the intervention groups remained significantly higher at all time points compared to pre-intervention, it is acknowledged that the mean scores started to decrease 3- and 6-month post-intervention, which is common in education interventions on sensitive topics (e.g., sex and breast cancer education) (83, 88). A modular structure with progressive information might be helpful to improve the long-term preservation of knowledge, adherence to health behavior, and stability of attitudes (89, 90). This is consistent with the data obtained in the focus groups suggesting a modular design for breast education, for example, “twice a year.”

As discussed above, whilst the breast education intervention improved adolescent girls' attitudes to breasts, the focus group participants suggested that past experiences (e.g., feeling uncomfortable in a mixed gender changing room) may impact attitudes and feelings at an older age. Introducing breast education in primary schools might reduce embarrassment and foster a more positive attitude to breasts and breast issues at a younger age. In addition, the focus group participants suggested it might be beneficial to teach boys about breasts. Future research is recommended to investigate whether teaching boys about breasts and breast issues in primary school settings would help normalize this topic for boys.

Peer-led education was suggested by the focus group participants as a strategy to teach girls about breasts and breast issues. The importance and effectiveness of peer-led education has been highlighted in education programmes on sensitive topics such as sex and HIV education (91–93), breast cancer and breast self-examination education (94, 95). Such an approach might be appropriate for delivering breast education in schools, particularly given the budget constraints and time pressures within schools.

On a few occasions, participants stated that they used their knowledge gained in the breast session to “teach others” about breasts, for example, their mothers, sisters, or cousins which is referred to as “intergenerational transmission of knowledge” (83). This evidence reveals that teaching adolescent girls about breasts could result in transmission of the knowledge, thus allowing women with limited health information sources to obtain new knowledge about breasts and promoting positive breast habits. It represents a potentially valuable and cost-effective tool for increasing knowledge of multiple breast topics and positive breast habits among women (83).

### Strengths and Limitations

This study had a large sample size with a low attrition rate, and the groups (schools) were recruited from both privileged and less-privileged areas in London, UK, which increases the generalizability of the study findings. The study did not assess differences in intervention effectiveness by individual-level socio-economic status, school-level variables, ethnicity, or biological age. It is recommended that future studies explore differences in intervention effectiveness accounting for these factors. No objective measures were collected to evaluate physical activity participation or ability to check breasts and bra fit. However, focus groups were conducted to provide further evidence and in-depth understanding of the impact of the intervention on breast habits to confirm the survey results.

## Conclusion

This study provides the first comprehensive evaluation of the impact of an education intervention on multiple breast topics not only on adolescent girls' breast knowledge, but also on attitudes to breasts and engagement with positive breast habits. The intervention was equally effective in two intervention groups of differing area-level deprivation and socio-economic status and significantly improved girls' breast knowledge, attitudes to breasts, and engagement with positive breast habits. These improvements were sustained over a 6-month period, establishing the longer-term impact of the breast education intervention. Focus group findings supported the quantitative results and provided in-depth insight into the positive impact of the intervention, and of adolescent girls' desire to receive breast education in schools. Breast education represents a powerful tool to address breast issues in adolescent populations, which can empower them to take responsibility for their breast health, that is a major practical benefit of the intervention. Incorporating breast education in schools' curriculum should be considered by policy makers. To educate and empower adolescent girls, it is recommended that a modular structure be adopted, with progressive information so that it can be tailored according to the age and breast needs of adolescent girls.

## Data Availability Statement

The raw data supporting the conclusions of this article will be made available by the authors, without undue reservation.

## Ethics Statement

The studies involving human participants were reviewed and approved by Ethics Committee at St Mary's University, Twickenham. Written informed consent to participate in this study was provided by the participants' legal guardian/next of kin.

## Author Contributions

NB, JW-S, and JS conceived the study idea. AO designed the study and collected the data. The first draft of the manuscript was written by AO. All authors commented on drafts of the manuscript and approved the final manuscript.

## Conflict of Interest

The authors declare that the research was conducted in the absence of any commercial or financial relationships that could be construed as a potential conflict of interest.
